# Targeting T-cell metabolism to boost immune checkpoint inhibitor therapy

**DOI:** 10.3389/fimmu.2022.1046755

**Published:** 2022-12-07

**Authors:** Haohao Li, Alison Zhao, Menghua Li, Lizhi Shi, Qiuju Han, Zhaohua Hou

**Affiliations:** ^1^ Institute of Immunopharmaceutical Sciences, School of Pharmaceutical Sciences, Shandong University, Jinan, Shandong, China; ^2^ Cleveland Clinic Lerner College of Medicine at Case Western Reserve School of Medicine, Cleveland, OH, United States; ^3^ Thoracic Service, Department of Surgery, Memorial Sloan Kettering Cancer Center, New York, NY, United States

**Keywords:** tumor microenvironment, T cell metabolism, immune checkpoint, immune checkpoints inhibitor, metabolic reprogramming

## Abstract

Immune checkpoint inhibitors (ICIs) have shown promising therapeutic effects in the treatment of advanced solid cancers, but their overall response rate is still very low for certain tumor subtypes, limiting their clinical scope. Moreover, the high incidence of drug resistance (including primary and acquired) and adverse effects pose significant challenges to the utilization of these therapies in the clinic. ICIs enhance T cell activation and reverse T cell exhaustion, which is a complex and multifactorial process suggesting that the regulatory mechanisms of ICI therapy are highly heterogeneous. Recently, metabolic reprogramming has emerged as a novel means of reversing T-cell exhaustion in the tumor microenvironment; there is increasing evidence that T cell metabolic disruption limits the therapeutic effect of ICIs. This review focuses on the crosstalk between T-cell metabolic reprogramming and ICI therapeutic efficacy, and summarizes recent strategies to improve drug tolerance and enhance anti-tumor effects by targeting T-cell metabolism alongside ICI therapy. The identification of potential targets for altering T-cell metabolism can significantly contribute to the development of methods to predict therapeutic responsiveness in patients receiving ICI therapy, which are currently unknown but would be of great clinical significance.

## 1 Introduction

Immune checkpoint inhibitors (ICIs) targeting PD-1/PD-L1 or CTLA-4 have shown promising therapeutic effects in the clinical treatment of advanced solid cancers, including melanoma, non-small cell lung cancer (NSCLC), renal cell carcinoma (RCC), urothelial carcinoma (UCC), and classical Hodgkin’s lymphoma. However, the overall response rate is still low, as only 15%–30% of patients with solid tumors respond to ICI treatment due to drug tolerance and side effects (1, 2). Therefore, finding combination therapy options to improve the efficacy of ICI treatment is a matter of urgency.

Nowadays, several biomarkers, such as PD-L1, mismatch repair deficiency (dMMR), microsatellite instability (MSI-H), and tumor mutational burden, have been utilized to predict ICI treatment response (3). Although these biomarkers improve the efficacy of ICI therapy to some extent, more reliable and accurate clinical predictors for response and outcome in the era of ICI therapy are lacking.

In recent years, metabolic reprogramming has been reported to play essential roles during T-cell differentiation, effector function, and memory response. Increasing evidence demonstrates that exhausted T cells experience metabolic insufficiency, which impairs effector immunity and causes poor responsiveness to ICI treatment (4). Based on this, ICI therapy combined with metabolic targeting has been found to alter T cell metabolism and improve ICI therapy response rate (5, 6). However, it is still unclear how the crosstalk between metabolic reprogramming and immune checkpoints works, and which metabolic pathways and their related elements can be effectively targeted to improve ICI therapy.

In this review, we highlight the metabolic alterations (glucose metabolism, lipid metabolism, and amino acid metabolism) of T cells in the tumor microenvironment (TME) and explore the crosstalk between immune checkpoint signaling and metabolism in T cells. We summarize the recent advances in combined T-cell metabolic targeting and ICI-based therapeutic strategies and discuss future directions in therapeutic development.

## 2 The characteristics of metabolic reprogramming of T cells in the TME

Metabolic reprogramming of T cells plays a crucial role in their fate. Resting naïve T cells utilize normal oxidative catabolism to maintain their physiological functions (7). Upon antigen recognition, TCR and CD28 activate downstream signaling pathways, leading to the transformation of resting naive T cells into effector T cells, with rapid proliferation and functional activation (8, 9). At the same time, in order to meet the nutritional needs of T cell activation, T cells enhance mitochondrial function by upregulating metabolic programming processes that drive a transition from catabolism to anabolic processes (10). Glycolysis and glutamine metabolism are enhanced to support effector function. Effector T cells are able to uptake high levels of glucose and amino acids by upregulating the expression of the glucose transporter GLUT1, glutamine transporter ASCT2, and sodium-coupled neutral amino acid transporters (SNATs) (11, 12). Concurrently, enzymes related to glycolysis and amino acid metabolism, such as HK2, PDK1, and LDHA, are also upregulated (13, 14).

However, metabolic reprogramming is also a hallmark of cancer progression (15). The nutrient-deficient TME and growing oxygen consumption by rapidly proliferating tumor cells drive the metabolic adaptability of CD8+ T cells. These CD8+ T cells have to adapt to the TME in an exhausted state, performing a switch from glycolysis to FAO, downregulation of glutaminolysis, reduction of mitochondrial biogenesis, and higher rate of ROS production (16). Lipid uptake and metabolism-related enzyme expression is increased in exhausted T cells. Among them, the overexpression of fatty acid translocase CD36 or Acyl-CoA synthetase long chain family member 4 (ACSL4) may lead to ferroptosis, a type of regulated cell death caused by accumulation of lipid peroxide (17–19). Cholesterol acyltransferase (ACAT) and 3-hydroxy-3-methylglutaryl-coenzyme A reductase (HMGCR) are involved in cholesterol metabolism, and have recently been reported to be related to T cell exhaustion (20, 21). In the TME, there are also a small number of memory T cells used in lipid oxidation as a primary energy source to support mitochondrial respiratory capacity (22). When encountering secondary antigens, memory T cells rapidly reactivate aerobic glycolysis to promote more robust effector function and proliferation (23).

It has been reported that a formidable obstacle to the effectiveness of immunotherapy is the metabolic constraints of the TME (24, 25). In the TME, the high metabolic activity of cancer cells and limited blood supply contribute to the scarcity of important nutrients and oxygen, causing tumor cells and immune cells, which have similar metabolic demands, to compete for nutrients and oxygen (26). Tumor cells are able to suppress antitumor immunity by competing and consuming essential nutrients or reducing the metabolic adaptability of tumor-infiltrating immune cells in other ways (5, 27). Therefore, metabolic intervention is expected to improve the effectiveness of immunotherapy. Nevertheless, this metabolic similarity also poses difficulties for metabolic intervention in the microenvironment. Since adoptive transfer technology is of great practical value as an effective approach for immunotherapy, targeting T cell metabolism may yield better outcomes in reversing T cell exhaustion and dysfunction.

## 3 Crosstalk between T cell metabolism and immune checkpoint signaling

Upregulation of immune checkpoint expression and metabolic reprogramming are both hallmarks of T cell exhaustion. The crosstalk between T cell metabolism and immune checkpoint signaling is complicated, but knowledge on these intricate associations provides the basis for rational design that enhances the feasibility of the combination of T cell metabolic targeting and immunotherapy.

### 3.1 Immune checkpoint signaling affects T cell metabolism

There is increasing evidence that checkpoint signaling could regulate metabolism of T cells (28, 29). CD28-mediated activation of the PI3K/Akt pathway promotes increased glucose uptake and metabolism in T lymphocytes, which could be blocked by PD-1 signaling or CTLA-4 ligation (30). Moreover, the T cell’s ability to absorb and utilize branched chain amino acids and glutamine is inhibited, while fatty acid oxidation is maintained by PD-1 signaling (28, 31). In contrast to PD-1, CTLA-4 inhibits glycolytic reprogramming without increasing the rate of fatty acid β-oxidation, thus preserving the metabolic profile of unstimulated T cells. This suggests that targeting T cell metabolism in combination with PD-1 blocking antibodies may achieve enhanced therapeutic efficacy (28).

Mitochondria are the hubs of cellular metabolism, including glucose, fatty acid, and amino acid metabolism (32). PD-1 signaling compromises oxidative phosphorylation in T cells by reducing mitochondrial fitness (29). Moreover, PD-1 stimulation causes severe structural and functional changes in mitochondria *via* downregulation of MICOS-associated proteins, which link the inner boundary to the outer mitochondrial membranes and stabilizes cristae junctions through various mechanisms, including a reduction in the number and length of mitochondrial cristae (33). Mechanistically, PD-1 inhibits the expression of Bhlhe40, which is a stress-responsive transcription factor that is important in maintaining mitochondrial fitness in TILs in the B16 melanoma model, inducing mitochondrial dysfunction (34). This evidence suggests that the mitochondria are another major target of the effects of PD-1 on cellular metabolism.

In addition to PD-1 and CTLA-4, other checkpoints also exhibit complex effects on T-cell metabolism. CD8+ T cells in gastric cancer highly express TIGIT, which blocks the metabolic pathway of CD8+ T cells. CD155-TIGIT signaling is reported to inhibit Akt and mTOR phosphorylation, glucose uptake, lactate production, and expression of the glycolytic enzymes GLUT1 and inhibiting hexokinase (HK1/HK2) in T cells (35). Tim-3 attenuates glucose uptake and consumption by inducing HK2 expression in macrophages through the STAT1 pathway and altering the expression of the glucose transporter GLUT1 in Jurkat T cells (36, 37). Although it has been previously reported that the ectopic expression of Tim-3 in T cells modulates the mTOR pathway, the mechanism remains to be clarified (38).

The above data suggest that the use of immune checkpoints appears to improve CD8+ T cell metabolism, but the triggers of impaired CD8+ T cell metabolism are multifaceted in the TME, and the use of immune checkpoint inhibitors alone cannot completely reverse impaired CD8+ T cell metabolism. Therefore, targeting their metabolism in combination with ICI therapy is a promising therapeutic option.

### 3.2 Metabolic reprogramming of T cells regulates the expression of immune checkpoints in the TME

Naive T cells express immune checkpoints such as PD-1 when the TCR signaling pathway is activated. When T cells are continuously stimulated, they become exhausted and continuously overexpress immune checkpoints. In addition, high levels of transforming growth factor β (TGF-β) and the angiogenesis-promoting molecule VEGF-A regulate the expression of immune checkpoint molecules on CD8+T cells in the TME (39, 40). Whether other mechanisms exist that induce immune checkpoint expression remains unclear and worthy of further exploration. Both metabolic reprogramming and upregulation of immune checkpoint expression are major hallmarks of T-cell exhaustion in the TME. There is now increasing evidence that metabolic reprogramming is positively and progressively associated with upregulation of immune checkpoint expression, rather than independent of each other, in tumor-infiltrating T cells.

Furthermore, the TME is rich in cholesterol. Cholesterol activates the endoplasmic reticulum stress sensor XBP1 through disruptions in lipid metabolism. XBP1 is a transcription factor that binds to the promoter of Pdcd1 and activates Pdcd1 gene transcription to induce CD8+ T cell exhaustion (21). However, Schmidt et al. found that elevated serosal cholesterol levels in CD8+ T cells increase TCR aggregation, thereby promoting immune synapse formation (41). Inhibition of cholesterol esterification enhances T cell effector function and antitumor response (41, 42). This paradoxical effect may be related to the variability of cholesterol levels in different disease models. As such, the role of cholesterol in CD8+ T cells still requires further elucidation.

Lactate (LA) in the TME may be related to the expression of PD-1 on CD8+ T cells (43, 44). CD8+ T cells survive poorly in an acidified environment (43). Under high-lactate conditions, lactate is taken up by CD8+ T cells through the monocarboxylate transporter (MCT1/4), and then oxidized to pyruvate *via* lactate dehydrogenase (LDH). Oxidized lactate ultimately disrupts the glycolytic pathway and dampens PD-1 expression (45). Conversely, regulatory T (Treg) cells rely on lactic acid as metabolic fuel, so high-lactate conditions enhance the ability of Treg cells to uptake LA from the microenvironment *via* MCT1 and metabolize it into phosphoenol pyruvate (PEP) (46, 47). PEP increases Ca^2+^ concentrations in the cytoplasm and promotes nucleocytoplasmic translocation of NFAT1 (46). NFAT1 positively regulates the expression of various immunological molecules, including PD-1 (48). It was recently reported that the balance of PD-1-expressing CD8+ T cells and Treg cells in the TME determines the clinical efficacy of the PD-1 blockade (49). Therefore, lactic acid may be a potential target in improving the efficacy of ICIs.

Similar to lactate, tryptophan and its metabolites also play different roles in CD8+ T cells and Treg cells. Tumor-repopulating cells (TRC) highly express the enzyme IDO1, leading to abundant kynurenine release. The aryl hydrocarbon receptor (AhR) is induced and activated by kynurenine that is taken up by CD8+ T cells, which binds to and upregulates PD-1. Thus, TRCs seem to drive the upregulation of PD-1 in CD8+ T cells through a transcellular Kyn-AhR mechanism (50). Such TRCs are a self-renewing, highly tumorigenic subpopulation of cancer cells that play a crucial role in the initiation, promotion, and progression of tumorigenesis (51). Tregs, activated in the presence of IDO1, upregulate FoxO3a and sequentially PD-1, and then elicit sustained suppression though the PD-1/PTEN feedback loop (52). A recent study showed that GTP cyclohydrolase 1 (GCH1) can induce PD-1 elevation in both Tregs and CD8+ T cells through a 5-HTP–AHR–IDO1-dependent mechanism (53). To this effect, multiple clinical trials have evaluated the combination of IDO1 inhibitors with immune checkpoint blockade agents, such as indoximod and epacadostat (54).

In conclusion, metabolic reprogramming of CD8+T cells in the TME may be an important cause of immune checkpoint upregulation. These results suggest that pharmacological inhibition of these pathways might also be an efficacious alternative strategy for targeting PD-1 in cancer.

### 3.3 Metabolic characteristics of patients predict the feasibility of immunotherapy

As described above, identifying the metabolic profile of ICI-responsive and non-responsive patients is essential in determining biomarkers that can predict ICI efficacy and metabolic targets that can be combined with immunotherapy.

A prospective clinical study shows that peripheral blood mononuclear cells (PBMCs) of melanoma patients who responded to PD-1 antibody had increased glycolysis, fatty acid metabolism, and tryptophan and branched chain amino acid metabolism, which supports increased mitochondrial function under stress (55). Moreover, analysis of CD8+ T cells shows that SLC2A14 and LDHC were highly expressed in the responder group (55). SLC2A14 and LDHC are genes encoding glucose transporter 14 (Glut-14) and lactate dehydrogenase C respectively, both of which are related to glycolysis (56, 57). These findings suggest that glycolytic signaling could serve as a predictive marker for ICI therapy and even a potential target for combination therapy. However, a study monitoring LDH in melanoma patients receiving anti-PD-1 treatment found that elevated LDH at baseline is associated with significantly shortened survival (58). The contradiction between these two studies may be related to the complexity of the TME, as the former only focuses on certain immune cells.

Another prospective clinical study shows that compared with serum samples from urological cancer patients who did not respond to nivolumab, the serum of responders was richer in long-chain fatty acids, and the very long-chain acyl-CoA synthetase SLC27A2 was more highly expressed in responders according to The Cancer Genome Atlas (TCGA) (59, 60). Moreover, Mock et al. hypothesized that the association of VLCFA-containing lipids with response is based on enhanced peroxisome signaling in T cells, which leads to a switch to fatty acid catabolism (59). The above results indicate that supplementation of VLCFA-containing lipids in patients with low serum levels of VLCFA or upregulation of SLC27A2 expression prior to the initiation of immunotherapy might help to achieve better therapeutic effect.

Other clinical studies show that in patients with different solid tumors treated with nivolumab, conversion of tryptophan to kynurenine increased, and that elevated levels of kynurenine are closely associated with their low survival rates (61, 62). This provides the basis for potential future avenues of exploration in the quest to reduce current therapeutic challenges related to drug tolerance during ICI treatment. Although there was no correlation between kyn/trp ratio and response in certain solid tumors (i.e. RCC, HNSCC), serum kyn/trp ratio still has both prognostic and predictive values in solid tumor patients treated with immunotherapy (62). The role of serum kyn/trp in immunotherapy should be further studied, and its role in the metabolism of immune cells, especially CD8+ T cells, should be explored as a potential immunotherapy target.

During tumor development, dysregulated metabolic programming leads to impaired T cell immune function, which is an important contributor to immunotherapeutic failure (63). Understanding the crosstalk between metabolic dysregulation and immunotherapy could reveal new therapeutic strategies to reactivate exhausted T cells and improve clinical outcomes of current ICI treatments.

In summary, analyzing the metabolic characteristics of immunotherapy responders and metabolic changes before and after treatment can help identify metabolic regulatory elements that may be potential therapeutic targets in combination with immunotherapy.

## 4 Targeting T cell metabolism to boost immune checkpoint inhibitor therapy

In the TME, T cells are often in a state of exhaustion, and immune checkpoint inhibition restores their effector functions to a certain extent. However, T-cell exhaustion is often multifaceted. We review current literature on metabolic targeting combined with ICIs in four categories: lipid metabolism, sugar metabolism, amino acid metabolism, and adenosine metabolism.

### 4.1 Lipid metabolism

Lipids are an essential component of biological membranes and play critical roles in energy supply and signaling for many cellular activities (64). Lipids are the main energy source for exhausted T-cells, and there is significant crosstalk between lipid metabolism and checkpoint signaling (28). Therefore, targeting lipid metabolism is expected to improve the efficacy of immunotherapy.

#### 4.1.1 Targeting lipid transport receptors could improve ICI response rate

**Figure 1:**
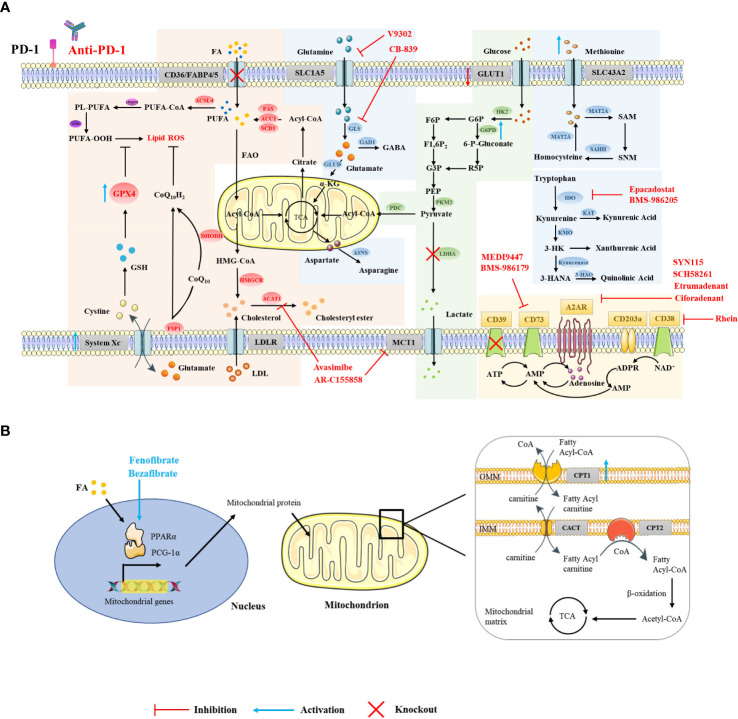
Strategies for targeting CD8+ T cell metabolism in combination with ICIs. Using small-molecule drugs or gene interference technology to target relevant elements in the metabolic pathways in the cytoplasm **(A)** and mitochondria **(B)** of CD8+ T cells, or targeting transcription factors that regulate metabolism **(B)**, can improve the efficacy of ICI therapy and exert a greater antitumor effect.

CD36 and FABP4/5 are major transport receptors mediating lipid uptake. Ma et al. found that cholesterol in the TME upregulates CD36 expression on CD8+ T cells, which enhances the uptake of polyunsaturated fatty acids and mediates lipid peroxidation, eventually leading to CD8+ T cell dysfunction. Additionally, targeting ferroptosis or CD36 was found to enhance the efficacy of CD8+ T cell- and ICI-based cancer immunotherapy (**Figure 1A**) (17). Mechanistically, Xu et al. found that excessive intake of oxidized low density lipoprotein by CD36 activates p38 kinase and its downstream signaling pathways, inducing death in CD8+ T cells (65). The inhibition of PD-1 and p38 signaling pathways together may enable the proliferation of the T_EMRA_ (effector memory T cells expressing CD45RA) subset (66). Similarly, our unpublished data also suggest that the use of sulfo-N-succinimidyl oleate (a CD36 inhibitor) can reduce the lipid peroxidation of antigen-specific CD8+ T cells and restore their effector function. Unlike CD8+T cells, it has been reported that CD36 maintains the mitochondrial fitness of Treg cells *via* the peroxisome proliferator-activated receptor-β signaling pathway in the TME (67). The different effects of CD36-mediated lipid uptake in the TME on different immune cells may be due to the different extents of their own lipid metabolism (68). Furthermore, other lipid transporters, such as FABP4/5, also play an assisting role in ICI therapy. PD-L1 blockade reduced FABP4/5 expression in tumor cells but increased FABP4/5 expression in Trm cells, providing adequate lipid uptake in Trm cells and contributing to antitumor immune response (69). These results indicate that appropriately increasing lipid uptake, rather than excessive lipid accumulation, contributes to improving the efficacy of ICI therapy.

#### 4.1.2 Targeting lipid peroxidation-related enzymes is a potential strategy to improve ICI therapy

Excessive lipid accumulation can induce lipid peroxidation and lead to ferroptosis (19). GPX4 can rescue cells from ferroptosis by degrading lipid peroxides (70). Although there is no direct evidence that there is crosstalk between GPX4 and immune checkpoints, many studies have shown that targeting ferroptosis in addition to ICI therapy can achieve better antitumor effects (17, 18). Whether the expression of GPX4 is related to the efficacy of immunotherapy still needs further exploration. The question of whether ferroptosis-related genes, i.e. GPX4, FSP1, DHODH, are able to boost immunotherapy, warrants further investigation. Although studies have shown that selenotherapy does not on an aggregated data level substantially affect the PD-1/PD-L1 axis, as determined by soluble PD-L1 analysis, there exists a dynamic change in individual soluble PD-L1 levels (71). Given that GPX4 is a selenoprotein, our unpublished data suggest that nutritional levels of seleninic acid can enhance the expression of GPX4 in antigen-specific CD8+ T cells and restore their IFN-γ and TNF-α production, giving rise to the possibility that select patients may benefit from combined GPX4 targeting and ICI therapy.

#### 4.1.3 Targeting lipid metabolism-related enzymes could help improve the efficacy of ICI therapy

Although the role of cholesterol on T cell metabolism in the tumor microenvironment is contradictory, targeting ACAT to inhibit cholesterol esterification and targeting HMGCR to inhibit cholesterol synthesis both reverse the exhausted state of T cells (41, 42). ACAT inhibition enhances responsiveness to PD-1 blockade in the setting of hepatocellular carcinoma (41). Targeting HMGCR may also enhance checkpoint-blocking therapy, since cholesterol induces increased expression of immune checkpoints and CD36 (21).

#### 4.1.4 Small-molecule compounds targeting lipid metabolism combined with ICI therapy improves the effector function of CD8+ T cells

Activated CD8+ T cells lacking both glucose and O_2_ enhance FA catabolism in the TME. PPARs are lipid sensors that modulate whole-body energy metabolism (72). Fenofibrate (FF) is a PPAR-α agonist that increases FA catabolism and preserves the effector functions of CD8+ TILs. FF-treated CD8+ TILs express increased PD-1 (73). Bezafibrate, an agonist of PGC-1α/PPAR complexes, promotes the FAO pathway by increasing the expression of CPT1, an enzyme key to fatty acid metabolism in the mitochondria (**Figure 1B**) (74, 75). The bezafibrate and PD-1 blockade combination activates mitochondrial biogenesis and FAO in CD8+ T cells, enhances survival and proliferation of tumor-reactive cytotoxic T lymphocytes (CTLs), and improves the efficacy of PD-1 blockade against unresponsive tumors with systemic immunosuppressive properties (75). In addition, direct activators of CPT1, PGC-1α (a regulator of mitochondrial biogenesis), and AMPK also synergize with PD-1 blockade therapy (75, 76).

### 4.2 Glucose metabolism

The effector capacity of cytotoxic T cells is tightly linked to their metabolic fitness, particularly to their glycolytic capacity (77). However, in the TME, glycolytically activated T cells often become anergic, leading to an increase in immune checkpoint proteins due to a lack of glucose and an unfavorable lactate gradient for release (9, 78). Next, we will explore the target selection in the glucose metabolic pathways of T cells that can be utilized to improve immunotherapy.

#### 4.2.1 Targeting glucose uptake may enhance ICI immunotherapy

As mentioned earlier, SLC2A14 in T cells was highly expressed in PD-1 blockade responders (55). Another study showed that there is lactate accumulation and upregulation of GLUT-1 in pancreatic ductal adenocarcinoma; high expression of GLUT-1 correlates with higher tumor grade and a higher density of PD-1^+^ T cells in human PDAC sections (78). This indicates that in the TME, both tumor cells and immune cells upregulate glucose transport receptor expression in order to compete for glucose energy supply, which is also the main factor underlying therapeutic inefficacy of immunotherapy. Therefore, impairing the glycolytic metabolism of tumor cells or improving the metabolism of immune cells can enhance the benefits of immunotherapy.

#### 4.2.2 Targeting glucose-metabolizing enzymes is also a potential strategy for combined ICI therapy

Clinical data show that increased numbers of CTLs expressing granzyme B are associated with better clinical outcomes in human mesothelioma and lung cancer patients treated with immune checkpoint blockade immunotherapy (79, 80). Activation of G6PD, a ‘metabolic checkpoint’ that controls glucose metabolic flux partitioning between aerobic glycolysis and the pentose phosphate pathway, increases Gzmb expression in tumor-specific CTLs (81). G6PD is therefore a potential molecular target for metabolic reprogramming of tumor-specific CTLs to improve immunotherapy.

#### 4.2.3 Lactate metabolism is closely related to the success of ICI therapy

Monocarboxylate transporter 1 (MCT1), highly expressed in Treg cells in the TME, mediates lactate uptake and induces PD-1 expression (46, 82). Inhibition of MCT1 in Treg cells significantly augments the antitumor efficacy of anti-PD-1 therapy (46). Activated T cells highly express lactate dehydrogenase A (LDHA) to support aerobic glycolysis to increase IFN-γ expression (83). However, when tumor cells overexpress LDHA, excess lactate is produced, the accumulation of which contributes to the creation of an acidic TME, impairing the antitumor function of T cells (84, 85). Furthermore, given that patients with dMMR tumors are likely to respond to treatment with ICIs, LDHA positively regulates MMR protein expression in dMMR and mismatch–repair‐proficient (pMMR) colorectal cancer, while LDHA inhibition can improve the efficacy of ICIs in pMMR colorectal cancer (86, 87). Therefore, selectively targeting LDHA is beneficial to improving ICI therapeutic efficacy and reversing T cell exhaustion. By promoting immunosuppression in the TME, LDHA is able to promote resistance to targeted therapy (88). Thus, targeting LDHA may be beneficial in improving immune tolerance to ICI therapy.

Certain costimulatory molecules, such as CD28, 4-1BB, and GITR, can enhance T cell activation and proliferation by upregulating glycolysis and enhancing fatty acid oxidation. Combined with immune checkpoint blockade therapy, these molecules can enhance the effector function of T cells and support a more powerful antitumor efficacy (89–91). Some immune checkpoint molecules can also affect T-cell metabolism. For instance, CD155 expressed in gastric cancer cells interacts with TIGIT, resulting in inhibition of glucose uptake and impaired T cell effector function (35). Therefore, the combined blockade of TIGIT and PD-1 elicits greater enhancement of immune activation (35, 92).

Current literature suggests that glycolysis can increase PD-L1 expression in tumor cells in addition to immune cells, and that highly glycolytic tumors respond more favorably to immunotherapy (93). These findings suggest that targeting glycolysis may be strategic in improving immunotherapy.

### 4.3 Amino acid metabolism

A variety of amino acids, including glutamine (Gln), arginine (Arg), and tryptophan (Trp), are key energy sources and substrates for protein and nucleic acid biosynthesis, and are therefore necessary for T cell activation, differentiation, and effector function (94, 95).

#### 4.3.1 Targeting amino acid uptake enhances ICI therapy

It is reported that activated T cells upregulate glutamine transporters (i.e. ASCT2, SNAT1, and SNAT2) to increase glutamine uptake and metabolism, thereby supporting proliferation and cytokine production (12, 96). Unfortunately, tumor cells are significantly more competitive for glutamine than T cells in the TME (97). High levels of the glutamine transporter SLC38A1 in tumor tissues are inversely proportional to CD8+ T cells, and there is a negative correlation between glutamine metabolism genes and markers of T cell-mediated cytotoxicity (98, 99). Other amino acids, such as cysteine, cystine, methionine, and alanine, have also been shown to enhance immunotherapy (100–102). For example, studies have shown that the number of infiltrating CD8+ T cells is inversely correlated with the expression of the cystine transporter system Xc- in human melanoma tissue, and the degradation of cystine and cysteine by cyst(e)inase combined with PD-L1 blockers can synergistically enhance T cell-mediated antitumor immunity and induce tumor cell ferroptosis (100, 103). Tumor cells consume more methionine than T cells through high expression of the methionine transporter SLC43A2, thus disrupting methionine metabolism in CD8+ T cells. Inhibition of tumor methionine uptake by knocking out SLC43A2, combined with anti-PD-L1 treatment, can enhance anti-tumor T cell responses and further inhibit tumor growth (101).

#### 4.3.2 Targeting amino acid metabolism-related enzymes is an effective means of improving ICI therapy

Tryptophan catabolism can mediate tumor immune escape through multiple pathways. For example, the accumulation of tryptophan-related metabolites kynurenine and 3-HAA can upregulate PD-1 expression in CD8+ T cells and impair T-cell function. Additionally, the aryl hydrocarbon receptor (AHR) promotes Treg differentiation, drives increased cellular expression of CD39 in macrophages, and reduces DC function, thereby inhibiting T cell activation (50, 95, 104). The upregulation of the tryptophan-kynurenine-aryl hydrocarbon receptor (Trp-Kyn-AhR) pathway, the major pathway for tryptophan catabolism, has been shown to be associated with impairment of antitumor immunity (105). Kynurenine-degrading enzyme and AhR inhibitors have demonstrated therapeutic activity as monotherapies, the effects of which are enhanced when in combination with anti-PD-1 agents (106, 107). Furthermore, glutamine metabolic enzymes, such as transglutaminase 2 (TG2), glutamate decarboxylase 1 (GAD1), and glutamine-dependent asparagine synthetase (ASNS), are also potential metabolic targets in combination with immune checkpoint therapy (95, 108). For arginine metabolism, Arg2 is highly expressed in Tregs, which weakens mTOR activity and enhances Treg inhibitory activity (109). However, tumor cells are also highly arginine-dependent, and many arginine-metabolizing enzymes can be co-expressed in various cells, leading to complex interactions (110, 111). Therefore, the mechanisms of arginine metabolism, as well as the sensitivity of tumor and immune cells to arginine, warrant further elucidation.

#### 4.3.3 Small-molecule compounds targeting amino acid metabolism combined with ICI therapy exert greater anti-tumor effects

The inhibition of glutamine utilization increases PD-L1 expression by reducing glutathione levels and inhibiting Sarco/ER Ca^2+^-ATPase (SERCA) activity, thereby impairing cytotoxic T cell activity (112). Therefore, combining glutamine utilization blockade with immune checkpoint blockade demonstrates synergistic antitumor effects (112). Furthermore, the use of glutaminase inhibitors, i.e., CB-839, in the TME inhibits the expansion and activation of CD8+ T cells (113). However, another glutamine transporter inhibitor, V-9302, selectively blocks glutamine uptake in triple-negative breast cancer (TNBC) cells but not CD8+ T cells, thereby improving CD8+ T cell effector function **Figure 1A** (99). Recent studies have shown that V-9302 enhances the infiltration and activation of CD8+ T cells by promoting autophagy in breast cancer cells, reducing B7H3 expression, and regulating the accumulation of reactive oxygen species (ROS). Additionally, V-9302 increases the antitumor activity of anti-PD-1 immunotherapy in breast cancer mouse models (114). However, contrary to the above experimental conclusions, Nabe et al. found that pretreatment with glutamine deprivation or inhibitors enhanced the transformation of T cells into a memory phenotype, showing decreased PD-1 expression and increased Ki67 positivity and thus providing one potential option for improving adoptive therapy (115). This suggests that different metabolic interventions given at different stages of CD8+ T cell differentiation influence their effector function. Indoleamine 2,3-dioxygenase 1 (IDO1) is upstream of the Trp-Kyn-AhR pathway and is the rate-limiting enzyme in tryptophan catabolism (116). In the Cancer Genome Atlas (TCGA) database, it has been observed that the gene expression of IDO1 is closely related to the expression of PD-1 (117). Despite this, some clinical trials have been terminated because of lack of clinical benefit, which may be due to compensatory mechanisms provided by tryptophan catabolism mediators such as TDO (Tryptophan-2,3-dioxygenase) and IDO2 (Indoleamine 2,3-dioxygenase 2), inadequate drug doses, insufficient duration of inhibition, and mismatched drug combination strategies. Improving these conditions by utilizing dual-targeted inhibitors, increasing dosages, and extending the duration or additional combinations of radiotherapy or chemotherapy, could be potential strategies for further clinical development (118, 119). In addition, CTLA-4 blockade combined with systemic inhibition of IDO1 can produce more therapeutically effective antitumor immunity than these interventions independently. This effect is T cell-dependent, resulting in increased infiltration of tumor-specific effector T cells and a significant increase in the proportion of effector to regulatory T cells in tumors (120).

#### 4.3.4 Exogenous amino acid supplementation is also one method of enhancing the efficacy of ICIs

Arginine restriction is a key feature of tumors, and the availability of L-arginine in the TME is a key determinant of effective antitumor T cell response (121, 122). A clinical study showed that T cells exhibit impaired proliferation, decreased IFN-γ release, and PD-1 upregulation in response to antigenic stimulation under low-arginine conditions (123). Oral L‐arginine boosts the antitumor effect of combination treatment with cyclophosphamide and anti‐PD‐1 antibody (124). Given the poor bioavailability of oral L-arginine, targeting arginase (Arg) or using arginine inhibitors can improve T cell proliferation and significantly enhance the antitumor effect of anti-PD-1 monoclonal antibodies and STING agonists (125). In addition, the combination of methionine supplementation and anti-PD-L1 can also increase T cell tumor infiltration and mediate synergistic antitumor effects (101).

Compelling evidence has shown that reprogramming amino acid metabolism is relevant in tumor immunotherapy. However, targeting is still an intractable problem. The development of targeted drugs or targeted intervention in amino acid metabolism can greatly improve the effect of tumor immunotherapy.

### 4.4 Adenosine metabolism

Adenosine is mainly produced from adenosine triphosphate (ATP) catabolism mediated by CD39 and CD73, and binds to adenosine receptors (AR) to trigger downstream signaling (126). Adenosine is higher in the TME, which induces accumulation of intracellular cAMP and impairs T cell-mediated antitumor responses (127). Several clinical trials demonstrated that targeting adenosine in combination with ICIs significantly increases the cytotoxic capacity of T cells (127).

#### 4.4.1 Targeting the CD39-CD73-adenosine metabolic pathway combined with ICI therapy exerts significant antitumor effects in various tumor models

CD39 and CD73 are highly expressed on Treg and tumor cells, leading to the accumulation of extracellular adenosine and thereby inhibiting the activation of effector T cells with up-regulated A2AR in the TME while enhancing the activity of Treg (128). CD73-expressing tumor cells are resistant to ICI therapy, and the dual blockade of CD73 and immune checkpoints improves the activation and effector function of antitumor T cells and increases the production of IFN-γ (129). Notably, CD73-targeting drugs combined with anti-PD-(L)1 therapy have achieved clinical efficacy, such as the anti-CD73 drug MEDI9447 combined with the anti-PD-L1 drug durvalumab in the treatment of advanced colorectal cancer or pancreatic cancer, or BMS-986179 combined with nivolumab in the treatment of various advanced solid tumors (**Figure 1A**) (130, 131). These studies show that blocking CD39 or CD73 can exert a synergistic antitumor effect in combination with ICI therapy.

#### 4.4.2 Targeting adenosine receptors combined with ICI therapy exerts greater antitumor effects

High concentrations of adenosine can induce T cells to overexpress adenosine receptors, which increases the expression of PD-1 in CD8+ T cells in the TME (129). In preclinical studies, it was found that although the use of A2aR inhibitors alone did not increase the frequency of antigen-specific CD8+ T cells, the dual blockade of PD-1 and A2aR significantly increased the proportion of tumor-infiltrating CD8+ T cells and the production of IFN-γ and Granzyme B and improved the survival rate of tumor-bearing mice (132, 133). In clinical studies, A2aR inhibitors combined with PD-1/PD-L1 blockers have demonstrated good therapeutic potential. For example, the A2aR small molecule inhibitor Ciforadenant combined with Atezolizumab improved T cell infiltration in patients with renal cell carcinoma resistant to PD-(L)1 antibody therapy **Figure 1A** (134). The dual A2aR/A2bR antagonist AB928 in combination with pembrolizumab significantly inhibited the growth of tumors in various tumor patients (135). In addition, the CTLA-4 or Tim3 blockade also exerted better antitumor effects in combination with A2aR blockade, although the underlying mechanism remains to be further elucidated (129, 136).

#### 4.4.3 Targeting the adenosine metabolic bypass pathway is a potential means of enhancing ICI therapy

The CD38 enzyme engages another adenosine production pathway independent of CD39 (137). The inhibition of CD38 expression on tumor cells reduces adenosine production and improves the efficacy of immune checkpoint therapy, demonstrating greater accumulation of CD8+ T cells and lower levels of Treg cells in mouse tumor models (138, 139). PD-(L)1 antibody treatment increased the expression of CD38 on tumor cells (138). In addition, CD38 is highly expressed on Treg cells, which are more immunosuppressive (140). CD38 mAb can inhibit Treg cells to overcome immunosuppression (140, 141). Moreover, PD-1^+^ CD38^hi^ CD8^+^ cells can be used as biomarkers of anti-PD-1 drug resistance (142). However, there is also evidence that CD38 is constitutively associated with lipid rafts, favoring the transduction of TCR signaling (143, 144). CD38 plays a complex role in cell metabolism and signal transduction, and whether targeting CD38 in the TME will affect the activation of T cells remains to be further studied.

Undoubtedly, targeting adenosine metabolism is currently the most widely studied therapy to boost immune checkpoint blockade. However, there are many mechanistic answers that need to be answered, such as how adenosine selectively inhibits the function of immune effector cells but enhances the activity of immunosuppressive cells and tumor cells. This may also be related to the variable expression of adenosine metabolic enzymes or receptors in different cell types.

## 5 Conclusion

In this review, we summarize the regulatory relationship of metabolism and T cell function and offer new insights into exploring potential trustworthy biomarkers. Furthermore, T cells undergo metabolic reprogramming in the TME, including a switch from glycolysis to FAO, the downregulation of glutaminolysis, and an increase in tryptophan and ATP catabolism, which critically contribute to T cell exhaustion and the futility of ICI therapy. We review the potential targets and mechanisms of action that target T cell metabolism to restore its function, providing a basis for clinical combinatorial therapies of ICI. Although some combination strategies have not yet been implemented clinically, others have gained promising results in pre-clinical studies. It is critical that further options for combination therapy are investigated.

Currently, anti-cancer immunotherapy with immune checkpoint inhibitors or adoptive cellular transfer have become the two most important approaches in cancer therapy. However, targeting remains an urgent problem to be further elucidated. Although targeting T-cell metabolism has yielded promising antitumor effects in *in vitro* studies, truly effective metabolic interventions that can work synergistically with immunotherapy still lag far behind clinical needs **Table 1**. Due to differences in cell metabolism and environments, the metabolic inhibition of tumor cells and the metabolic recovery of T cells are still challenging to implement. As adoptive cellular transfer technologies develop, targeting T cell metabolism to recover its function and utilizing efficient combinations of immune checkpoint inhibitors will be critical areas of exploration.

**Table 1:** Current research progress of CD8+ T cell metabolic targeting combined with ICIs therapy.

Metabolic agent	Immune-checkpoint inhibitor combination partner	Cancer types	Status	Effects	Reference
***Targeting lipid metabolism***					
CD36 gene knockout	InVivoPlus anti-mouse PD-1 (clone RMP 1-14)	Melanoma	Animal experiments	Extends mouse survival	(17)
Avasimibe (ACAT inhibitor)	Nivolumab	HBV-related HCC	Cell experiment	Enhances responsiveness to PD-1 blockade	(41)
Fenofibrate (PPAR-α agonist)	α-PD-1 Ab (clone 29F.1A12)	Melanoma	Animal experiments	Improves CD8+ TIL functions and works in synergy with PD-1 blockade to delay tumor growth	(73)
Bezafibrate (PGC-1α/PPAR agonist)	Anti–PD-L1 (clone 1-111A.4)	Colon carcinoma	Animal experiments	Combination therapy enhances survival and proliferation of tumor-reactive CTLs.	(75)
***Targeting glucose metabolism***					
AR-C155858 (MCT1 inhibitor)	Nivolumab	Colon carcinoma	Animal experiments	Decreases the frequency of Treg cells and PD-1 expression by Treg cells and increases activated CD8+ T cells in the intrahepatic TME	(46)
LDH-A knockdown	Anti-PD-1 mAb (Bioxcell, Lebanon, NH, USA)	pMMR colorectal cancer	Animal experiments	LDH-A inhibition can improve the efficacy of PD-1 blockade in a pMMR CRC xenograft model.	(87)
***Targeting amino acid metabolism***					
CB-839 (Glutaminase inhibitor)	InVivoPlus anti-mouse PD-1 (clone RMP 1-14)	KRAS-mutant lung adenocarcinoma	Animal experiments	Inhibits clonal expansion and activation of CD8+ T cells	(113)
V9302 (Glutamine metabolism inhibitor)	InVivoPlus anti-mouse PD-1 (clone RMP 1-14)	Breast cancer	Animal experiments	Strengthens CD8+ T cell infiltration and activation, and sensitizes breast cancer to PD-1 blockade therapy	(114)
Epacadostat (IDO1 inhibitor)	Pembrolizumab	Melanoma	Completed/ Phase 3	Does not improve progression-free survival or overall survival	NCT02752074
	Ipilimumab	Melanoma	Terminated/ Phase 1/2	Demonstrates clinical and pharmacologic activity and is well-tolerated in patients with advanced melanoma	NCT01604889
	Pembrolizumab	Neck Squamous Cell Carcinoma	Active/ Phase 2	Unknown	NCT03823131
	NCT03832673, NCT04463771, NCT03589651, NCT04586244				
***Targeting amino acid metabolism***					
BMS-986205 (IDO1 inhibitor)	Nivolumab	Muscle-invasive bladder cancer	Recruiting/ Phase 3	Unknown	NCT03661320
	Nivolumab	Endometrial Adenocarcinoma	Active/ Phase 2	Unknown	NCT04106414
	NCT03519256, NCT03854032, NCT03695250, NCT03792750, NCT03459222 …				
L-arginine supplementation	Anti-mouse PD-1 (clone RMP 1-14)	Colon carcinoma	Animal experiments	Increases the proportions of tumor peptide-specific CD8+ T cells in draining lymph nodes and increases the number of cured mice that are treated with CP and anti-PD-1 antibody	(124)
Methionine supplementation	Anti-mouse PD-L1 (Clone: 10F.9G2)	Ovarian cancer Melanoma	Animal experiments	Increases T cell tumor infiltration and mediates synergistic antitumor effects	(101)
***Targeting adenosine metabolism***					
Anti-mouse CD73 mAb (clone TY/23)	Anti-mouse PD-1 mAb (clone RMP1-14) Anti-mouse CTLA-4 mAb (clone 9H10 and UC10-4F10)	Colon carcinoma Prostate cancer Breast carcinoma	Animal experiments	CD73 blockade enhances anti-PD-1/CTLA-4 mAb therapy *via* IFN-γ and CD8+ T cells	(129)
MEDI9447 (CD73 ectonucleotidase activity inhibitor)	Anti-PD1 antibody (AMP-514)	Colon carcinoma Breast carcinoma	Animal experiments	Increases the number of CD8+ effector cells and activated macrophages	(130)
BMS-986179 (Anti-CD73 antibody)	Nivolumab	Malignant Solid Tumor	Completed/Phase 1/2	A total of 59 patients received bms-986179 monotherapy or combination therapy with nivolumab, of which 7 patients achieved partial response and 10 patients achieved stable disease. The safety of combination therapy was consistent with nivolumab monotherapy.	NCT02754141
SCH58261, SYN115 (A2AR antagonist)	Anti-mouse PD-1 mAb (clone RMP1-14)	Breast carcinoma	Animal experiments	Combination of PD-1 and A2A blockade significantly enhances the IFNγ production of tumor-infiltrating CD8+ T lymphocytes	(132)
Ciforadenant (A2aR inhibitor)	Atezolizumab	Renal Cell Cancer	Completed/Phase 1	Increases recruitment of CD8+ T cells into the tumor and broadens the circulating T-cell repertoire.	NCT02655822
Etrumadenant (Dual adenosine receptor antagonist)	Zimberelimab	Renal Cell Cancer	Recruiting/ Phase 2	Unknown	NCT05024097
Rhein (CD38 inhibitor)	Anti-PD-L1 (clone 9G2)	Lung cancer	Animal experiments	Increases activated CD8+ T cells and memory CD8+ T cells and substantially reduces primary tumor burden and metastases	(138)

The metabolic mechanisms of T cells have important implications for the therapeutic efficacy of ICI therapy. Therefore, metabolic alterations in T cells may correlate with different degrees of clinical responsiveness to ICI therapy. Therefore, further exploration of T cell metabolism and its regulators, together with more specific elucidation of metabolic differences in CD8+ T cells in cancer patients responsive to ICI therapy, is essential for the consideration of immune cell metabolism as part of assessing the clinical efficacy of ICI therapy. This review explores the correlation between T cell metabolic reprogramming and poor response to ICI therapies from the perspective of impaired T cell metabolism in the TME; potential combinatorial strategies to reinvigorate ICI therapies by targeting T cell metabolism-related molecules are enumerated to provide new ideas for the clinical selection of rational combinatorial strategies.

## Author contributions

HL, ML, and LS conceived of the manuscript. HL wrote the manuscript and prepared the figures. QH and ZH reviewed the manuscript and provided important information for the completion of this manuscript. All authors listed have made a substantial, direct, and intellectual contribution to the work and approved it for publication.
